# How to deal with negative preferences in recommender systems: a theoretical framework

**DOI:** 10.1007/s10844-022-00705-9

**Published:** 2022-04-26

**Authors:** Federica Cena, Luca Console, Fabiana Vernero

**Affiliations:** Department of Computer Science, University of Turin, C.so Svizzera 185, Turin, 10149 Italy

**Keywords:** Knowledge based systems, Negative feedback, Recommender systems

## Abstract

Negative information plays an important role in the way we express our preferences and desires. However, it has not received the same attention as positive feedback in recommender systems. Here we show how negative user preferences can be exploited to generate recommendations. We rely on a logical semantics for the recommendation process introduced in a previous paper and this allows us to single out three main conceptual approaches, as well as a set of variations, for dealing with negative user preferences. The formal framework provides a common ground for analysis and comparison. In addition, we show how existing approaches to recommendation correspond to alternatives in our framework.

## Introduction

Recommenders are systems capable of assisting users in finding relevant information or relevant services, thus helping them to cope with information overload (Burke, 2002). Recommendation relies on user preferences and, in particular, is usually based on positive preferences, i.e., on what the user likes, or in other words, a greater liking for one alternative over another or others (Chen & Pu, 2004). A preference is usually determined by multiple decision attributes. Multi-attribute utility theory deals with the problem where a decision maker chooses from a number of alternatives that he/she evaluates on the basis of two or more criteria. Normally, the decision maker aims to maximize his/her utility function (Dyer et al., 1992). Many methods have been developed to model a decision maker’s multi-attribute utility function that can represent his/her complete preference (Pomerol & Barba-Romero, 2000).

Instead, the aim of this paper is to focus on negative preferences (i.e., what the user does not like) and to analyse how they can be exploited in the recommendation process. Negative information plays an important role in the way we express our preferences and desires. In everyday life it is very common to specify what we do not like or even what we hate whenever we have to choose something to do or whenever someone asks us what we would like to do. For example, when asked about food, people usually specify what they do not like or what they would not want to eat. In some cases, a negative feedback may even have a greater importance for a user than a positive one. Some psychological studies demonstrate that not only emotionally negative experience has a stronger impact on an individual’s memory (Kensinger, 2009), but it also has a greater effect on human behavior in general (Rozin & Royzman, 2001). This is known as the negativity bias. Similarly, according to the loss aversion theory (Kahneman & Tversky, 2013), people value loss and gain differently - the pain from a loss is psychologically more powerful than pleasure from a similar gain. For instance, the feeling of disappointment over a poor book recommendation can outweigh the satisfaction provided by a good one. This is especially true for non-neurotypical users (such as people with autism, mental problems, anxiety, phobias etc), for whom negative experiences can cause severe consequences on the level of stress they have to endure (Hobson, 2019).

Another important aspect of negative preferences regards recommendation informativeness and transparency. High transparency can help to build trust in the system, which might be particularly important for new users. Highlighting negative recommendations can help to strengthen user trust when only few positive recommendations are available.

Therefore, to improve users’ experience and their trust in the system, it is important for recommender systems to distinguish between positive and negative user preferences -and to exploit both of them. However, negative information has not received the same attention as positive one so far. In fact, most existing recommender systems produce personalized rankings for users by differentiating previous positive choices from all other choices, while negative preferences are disregarded. These algorithms only optimize the benefit of recommending positive items, and ignore the cost of negative items as they are considered similar to unknown items.

One reason for this choice is the fact that information about negative preferences is less commonly available to recommender systems. Firstly, negative preferences are harder to acquire through implicit channels (Gauch et al., 2007) because users mainly pursue information they consider as interesting. Some systems try to infer negative preferences from user behaviour, for example, from the fact that users do not bookmark (Lee & Brusilovsky, 2009) or do not select some recommended items (Peska, 2017; Zhao et al., 2018), while others take into account different actions (page view, scroll, time on page) and consider as negative all feedback below a certain threshold (Peska & Vojtas, 2013). Moreover, sentiment analysis has been used to implicitly infer negative opinions from explicitly-expressed user comments, see for example (Zhang et al., 2015; Musto et al., 2017; Ziani et al., 2017). For an extensive review on preference elicitation methods in recommender systems see (Atas et al., 2021; Chen & Pu, 2004; Huang, 2011; Knijnenburg & Willemsen, 2009; 2010).

Secondly, recommender systems that draw information from user ratings usually allow to express different levels of preference, but rarely offer the possibility to provide truly negative ratings. In fact, it can be inappropriate to explicitly ask the user to express negative feedback, which, in some cases, could favour hate speech. In traditional collaborative filtering approaches (Sarwar et al., 2001), user preferences for new items are predicted based on the user’s previous ratings and on the opinions of other like-minded users. In this way, negative preferences are taken into account at least implicitly, but they do not enjoy any special status. Only few systems, such as those described in (McCarthy & Anagnost, 1998; Chao et al., 2005; Hamed et al., 2012), proactively ask users to express negative preferences. Notably, however, Social Recommender Systems and other commercial platforms such as YouTube^1^, allow explicitly negative ratings, and even Facebook^2^ provides some way to express discontent with regards to the posts of other users, even if only in the form of “sadness” or “anger”.

In addition, standard recommender models are usually unable to properly operate with negative feedback and more often ignore it (Frolov & Oseledets, 2016). As an example, a well known library for recommender systems, MyMediaLite (Gantner et al., 2011), that features many state-of-the-art algorithms, does not support negative feedback for item recommendation tasks. (Frolov & Oseledets, 2016) demonstrated that standard collaborative-based recommendation algorithms are unable to accurately predict relevant items given only an example of user preferences with low ratings. Also in content-based systems, negative preferences are hardly ever exploited since traditional techniques cannot handle them: for example, (Musto et al., 2011) needed to enhance a traditional vector space model to enable it to incorporate negative user preferences. Indeed, a number of methodologies have been proposed for traditional techniques to deal with the problem of considering negative preferences (Frolov & Oseledets, 2016). For example, given an item with a negative feedback, algorithms can look for the least similar items. However, it is not even clear when to switch between the “least similar” and the “most similar” recommendation modes, especially when the rating scale to collect user evaluations is only positive.

In this paper we aim at raising awareness of the role of negative preferences in recommender systems. More specifically, we focus on how negative information can be used in the recommendation process and how it can provide significant benefits. In particular, we will show that negative user preferences can be exploited in different ways. In order to provide a common background to characterize these different conceptual approaches we will specifically focus on knowledge-based recommender systems and adopt a logical characterization of the recommendation process that we introduced in a previous paper (Cena et al., 2021). Such logical characterization endows the whole recommendation process with a precise semantics, in accordance with the growing demand for transparency and explainability in the explainable Artificial Intelligent vision (Došilović et al., 2018; Tintarev & Masthoff, 2015).

We will single out three main conceptual approaches for dealing with negative user preferences (and a set of variations of these approaches). Our formal framework will offer a common ground to compare the approaches, allowing us to highlight similarities and differences among them in a clear way. In the previous paper we argued that the adoption of a logical framework (which we borrowed from the formalization of other problem solving tasks such as diagnosis (Hamscher et al., 1992)) allowed us to analyse a recommendation generation task at a conceptual level, disregarding the details of specific algorithms and implementations, and to compare them at a conceptual level. Similarly, in this paper we aim at analysing the different roles that negative preferences can play in this problem solving task. In the second part of the paper we discuss how a number of recommenders in the literature map to our framework, which will thus be used as a reference for classification. This will also allow us to point out that there are opportunities offered by negative preferences which have not been extensively exploited in recommenders so far.

The main contributions of the paper are the following:

- We raise awareness of the role and possible benefits of negative preferences;
- We systematically study the use of negative preferences in the recommendation process;
- We provide a formal framework that offers a common ground to compare existing approaches, allowing to clearly highlight similarities and differences among them.

The paper is organized as follows. In Section 2, we summarize the logical characterization of the recommendation process presented in (Cena et al., 2021). In Section 3, we show how negative preferences can be exploited either to generate negative recommendations (Section 3.1), or to impose constraints on positive recommendations (Section 3.2), or, finally, to express approximate positive preferences (Section 3.3). In Section 4 we compare the different approaches, while in Section 5 we classify state-of-art work with respect to the different ways of dealing with negative preferences we have highlighted. Section 6 presents a qualitative evaluation aimed at assessing how users perceive recommendations based on negative preferences in comparison with recommendations which are unaware of them. Finally, we discuss our framework in Section 7.

## A logical semantics for recommender systems

This section summarizes the logical characterization of recommendation that we introduced in (Cena et al., 2021) which will provide a formal, yet simple, formalism that allows us to focus on the conceptual aspects of dealing with negative information, avoiding all the details of specific approaches and implementations of recommendation. In other words, logic is used as a neutral formalism which allows us to highlight how negative information can contribute to provide better recommendations to a user. The logical semantics can then be implemented in different ways in a recommender.

Borrowing from previous characterizations of other problem solving tasks (and most notably diagnosis (Hamscher et al., 1992)), we started from a very general definition of knowledge-based recommendation problem. The knowledge base of a recommender is made of three main ingredients:

- A vocabulary of features concerning the user and her/his preferences;
- A vocabulary of features concerning the context;
- A vocabulary of features concerning the items to be recommended;

The knowledge base includes relations among these features (in the paper we abstract from the source of such a knowledge base, which could be provided by an expert or could be learnt from examples; this is irrelevant in all formalization that follows which is independent of this issue). The relations will be represented as logical formulae according to the pattern

### features of the ītem to be recommended and of the context → features of the user

specifying that an item to be recommended is suitable, in a given context, for some features concerning the user and her/his preferences. More formally:

### Definition 1.

A **recommendation problem** *RP* is a pair *RP* = 〈*DM*, *OBS*〉, where:

- *DM* is a set of logical formulae, involving the following set of predicates:
  - *F* is a set of predicates representing user features and preferences,
  - *R* is a set of predicates representing items among which the one(s) to be recommended have to be chosen,
  - *C* is a set of predicates representing contextual features.
- *OBS* = 〈 UF, CXT 〉 is a set of ground atoms denoting the case to be solved and
  - *UF* are ground atoms representing features and preferences of a specific user,
  - *CXT* are ground atoms representing a specific contextual situation.

*DM* is the set of formulae constituting the recommender knowledge base and following the above-discussed patterns, i.e., $t \equiv t_{1} \rightarrow t_{2}$, where *t*_1_ involves features in *R* and *C* and *t*_2_ involves features in *F*.

As a running example throughout the paper, we take inspiration from an existing recommender system, iCity (Carmagnola et al., 2008), that suggests cultural events that take place in the Municipality of Turin, Italy. iCity exploits an overlay model over an ontology to represent the domain. Applying our formalization to this system, we have for example the following recommendation problem, where we consider the following sets of predicates (the example is propositional to simplify the discussion):

- *F* = {= *age*(*X*) −−*whereXcanassumethemutuallyexclusivevalues* : *children*, *young*, *adult*, *middle*_*aged*, *elder*, *budget*(*X*) − *whereXcanassumethevalues* : *low*, *medium*, *high*, *interests*(*X*) − *whereXcanassumethevalues* : *music*, *food*, *history*, *art*, *wine*..., *food*_*preferences*(*X*) − *whereXcanassumethevalues* : *pizza*, *fastFood*, *vegan*, *meat*, *fish*, *low*_*calorie*, *alchoolics*,...}
- *R* = {= *concert*, *restaurant*, *exhibition*, *museum*, *japanese*_*restaurant*, *chinese*_*restaurant*, *wineBar*, *rock*_*concert*, *jazz*_*concert*,...}
- *C* = {*device*(*X*) − *whereXcanassumethevalues* : *desktop*, *mobile*), *time*(*X*) − *whereXcanassumethevalues* : *morning*, *afternoon*, *evening*, *night*},

The domain model *DM* contains formulae linking F and C with R, such as:

*DM* = $\{ concert \rightarrow age(young) AND interests(music)$, $museum AND (time(morning) OR time(afternoon) \rightarrow $ *age*(*middle*_*aged*)*ANDinterest*(*art*), $exhibition \rightarrow interests(art),$ $pizza \rightarrow age(young),$ $fastFood \rightarrow age(young),$ $wineBar \rightarrow age(middle\_aged),$ $restaurant \rightarrow interest(food) AND age(adult),$ $wineBar \rightarrow interest(food) AND interest(wine)$, $pizza \rightarrow interest(food)$, …}

Solving a recommendation problem consists in finding a set of recommendation items (atoms of predicates in *R*) that are in accordance with the set of atoms in *OBS*, or, more specifically, that are in accordance with *UF*, given *CXT*. The notion of *being in accordance* can be formalized in a strong way as implication, leading to an abductive definition of recommendation (the recommended items must entail the user preferences) or in a weaker way as consistency (the recommended items need only be consistent with the user preferences). In the general definition that follows user preferences are partitioned into two subsets and a solution must entail the atoms in one of the subsets, while being consistent with those in the other subset.

### Definition 2.

**[Parametric solution.]** Given a recommendation problem *RP* = 〈*DM*, *OBS*〉, where *OBS* = 〈*UF*, *CXT*〉. Let us consider a partitioning of *UF* in two subsets *UF*^*A*^ and *UF*^*C*^. A set *S* of ground atoms of predicates in *R* is a solution to *RP* if and only if

- *DM* ∪ *S* ∪ *CXT*⊧*UF*^*A*^
- *DM* ∪ *S* ∪ *CXTisconsistentwithUF*^*C*^.

The definition is parametric with respect to the partitioning of *UF*: at one extreme there is the case where *UF*^*A*^=*UF*. This corresponds to a strong notion of recommendation where *in accordance with* means *entails* (*abductive definition*). At the other extreme there is the case where *UF*^*C*^=*UF* and *UF*^*A*^ = *∅*. This corresponds to a weak notion of recommendation where *in accordance with* means *consistent with*. In between there is a lattice of alternatives depending on the set *UF*^*A*^. The bottom of the lattice corresponds to the consistency-based definition (*UF*^*A*^ = *∅*); the top to the abductive definition (*UF*^*A*^=*UF*); all the intermediate cases correspond to selecting subsets of *UF* as *UF*^*A*^ and are partially ordered according to the subset relation. Thus we have a full spectrum of alternative definitions.

Let us consider the running example above. Given the observations *OBS*:

*UF* = {*interests*(*music*),*age*(*young*)}

*CXT* = {*time*(*evening*)}

According to Definition 2 (Parametric solution) and requiring that all user preferences are entailed, an abductive solution is: *S* = {*concert*}.

Notice that if we assume consistency in Definition 2, then also {*exhibition*} {*pizza*} and {*fast*_*food*} would be solutions as they do not generate inconsistencies with user preferences).

In the previous paper (Cena et al., 2021) we analyzed the spectrum of alternative definitions, comparing them and discussing criteria to select the most appropriate one, given a specific recommendation problem.

We also discussed the many opportunities which arise from the transposition of all the results achieved in the area of diagnosis to the recommendation problem (e.g., criteria for ranking and discriminating solutions, properties of the solutions, efficient approaches to compute them), thanks to the formal characterization, which thus provides a unified semantics for a varieties of approaches to recommendation.

In this paper we will show that the same framework can be used to analyse the role of negative user preferences. In particular, our formal semantics will allow us to single out a number of different conceptual ways of dealing with such type of information and will allow us to compare them and to provide guidelines for choosing among them.

## Dealing with negative preferences

In this section we discuss different ways of dealing with negative user preferences, i.e., information on what the user dislikes or, even strongly, hates. We will thus extend definition 2 (Parametric solution) in different ways to deal with such information.

At an abstract level we can single out three different ways of dealing with negative user preferences. They can be used:

- To provide negative recommendations, i.e., items to be avoided.
- As constraints, i.e., recommended items must not involve them.
- As approximate ways to express positive preferences.

The first approach corresponds to a common attitude we have towards what we don’t like: explicitly excluding the choices that are related to what we do not like. For example, if we do not like fish, then when we have to choose a restaurant we exclude fish restaurants from the choice. If we have to communicate this choice we would tell someone else something like “... let’s avoid fish restaurants, please ....”. Thus, we would expect this kind of negative recommendation also from an intelligent recommender system.

The second approach corresponds to the use of negative preferences as filters: once we have recommendations for positive preferences, we exclude those that involve also negative ones. As we shall see this notion of filtering can be defined in different ways, starting from strong logical filters to a variation of weaker filters that weigh recommendations according to how they involve positive and negative preferences. The main difference with the previous approach is that it does not mention explicitly what has to be avoided and in this sense it is less informative for the user.

The third approach takes the move from the idea that in some cases we use negative statements as an economic way of expressing disjunctive positive preferences or, said differently, of expressing indifference towards a set of positive possibilities. For example, if when choosing a restaurant we are indifferent to any type of food but fish, we would commonly express this preferences as “... any food is ok but fish ...”, rather than saying “pizza is ok or pasta is ok or meat is ok, ...”. Thus, in this case, negative preferences are actually a way of expressing positive preferences.

The next subsections analyse these three cases. We assume that user data *UF* is partitioned in a pair 〈*UF*^+^, *UF*^−^〉, where the former contains positive user preferences and the latter contains negative user preferences.

### Negative recommendations

A first approach to deal with negative preferences is to interpret them in a strong way as something that the user hates. In this case not only should the recommender system avoid recommending items which are in accordance with these preferences, but it should also explicitly provide negative recommendations about these items, that is, it should warn the user about the items to be avoided. Thus, in this case, a solution should contain two parts: (i) the positive recommendations, i.e., the items suggested to the user given her preferences and (ii) the negative recommendations, i.e., the items to be avoided. In logical terms, the solution to a recommendation problem should therefore include two sets involving recommendation items: a set of positive items which must imply (or be consistent) with the positive preferences and a set of negative items, which are the negation of those that imply the negative preferences:

#### Definition 3.

**[Negative recommendations.]** Given a recommendation problem *RP* = 〈*DM*, *OBS*〉, where *OBS* = 〈*UF*^+^,*UF*^−^,*CXT*〉. Let us consider a partitioning of *UF*^+^ in two subsets *UF*^*A*^ and *UF*^*C*^. The pair 〈*P*, *N*〉 of ground formulae in *R* is a solution to *RP* if and only if

- *DM* ∪ *CXT* ∪ *P* ∪ *N*⊧*UF*^*A*^
- *DM* ∪ *CXT* ∪ *P* ∪ *NisconsistentwithUF*^*C*^.
- *N* is the negation of the items that imply negative preferences, that is:
  - *N* = ¬*N*_1_ ∧¬*N*_2_ ∧… ∧¬*N*_*j*_
  - where for each *g*_*i*_ ∈ *UF*^−^ we have that *DM* ∪ *CXT* ∪ *N*_*i*_⊧*g*_*i*_

In other words, a solution involves the items which cover the positive preferences and the negation of the items which imply negative preferences. A solution can be computed in three steps:

1. Compute each positive recommendation *P*_*i*_ using positive preferences;
2. Compute each negative recommendation *N*_*j*_ using the negative preferences and then build $N_{j}^{-}$ as the set of the negations of *N*_*j*_;
3. Compute each solution as the merge $P_{i} \cup \neg N_{j}^{-}$ keeping those that are consistent.

We claim that this interpretation of negative preferences is the one that corresponds to the situation where users provide strong statements involving negative preferences to express *hate*, that is something that they definitely would not accept. Providing negative recommendations is the answer to this strong information and allows users to accept more easily and in a more secure way the positive recommendations, as they can be sure that what they hate is avoided. In other words, nothing is implicit and everything is disclosed: both what is appropriate and what must be avoided. Thus, in a sense, the solution is very informative for the user.

Given the example model in the previous section, let us suppose that we have the following user preferences

〈*UF*^+^ = {*age*(*young*)}, *UF*^−^ = {*interests*(*music*)}〉

Then we have two solutions:

*S*_1_ = 〈{*pizza*},{¬*concert*}〉 *S*_2_ = 〈{*fastFood*},{¬*concert*}〉

which mention the explicit negative recommendation of avoiding concerts.

Multiple solutions can be ranked based on the information content they provide to a user. Various criteria can be adopted; in particular one can compare the set of atoms that occur in solutions (looking at both the positive and the negative ones) and prefer the solutions that involve maximal sets of atoms (considering either superset or cardinal maximality). This is clearly a partial order; for example, the two solutions above cannot be ranked.

### Negative preferences as constraints (or filters)

In this case the idea is to use negative preferences as constraints on the solutions, in the sense that not only should the recommended items be consistent with positive preferences, but also with the negative ones.

We can reformulate the notion of solution to a recommendation problem.

#### Definition 4.

**[Negative constraints.]** Given a recommendation problem *RP* = 〈*DM*, *OBS*〉, where *OBS* = 〈*UF*^+^,*UF*^−^,*CXT*〉. Let us consider a partitioning of *UF*^+^ in two subsets *UF*^*A*^ and *UF*^*C*^ and let *NEG* be the set of the negations of the negative preferences in *UF*^−^. A set *S* of ground instances of predicates in *R* is a solution to *RP* if and only if

- *DM* ∪ *CXT* ∪ *S*⊧*UF*^*A*^
- *DM* ∪ *CXT* ∪ *SisconsistentwithUF*^*C*^ ∪ *NEG*.

Although it does not mention what has to be avoided, the consistency check in the second item of the definition guarantees that negative preferences are taken into account avoiding those positive recommendations that are in conflict with negative user preferences.

The definition in the previous section is preferable in case the user can express mainly negative preferences and few positive ones. In this case, in fact, definition 3 (Negative recommendations) can inform the user on what has to be avoided, while definition 4 (Negative Constraints) is less useful since it does not provide any information on the filtered out options, thus offering little guidance to the user. At the extreme case where only negative preferences are available, only definition 3 (Negative recommendations) can be useful. On the other hand, when many positive preferences are available, definition 3 (Negative recommendations) could be perceived as less useful by users, since they are already provided with a large set of positive recommendations to deal with. Notice that the same criteria discussed in the previous section can be adopted for ranking solutions.

Let us consider the example above and consider the user preferences:

〈*UF*^+^ = {*age*(*young*)}, *UF*^−^ = {*interests*(*music*)}〉

Then we have two solutions:

*S*_1_ = {*pizza*} *S*_2_ = {*fastFood*}

In this case the solutions are the same as those in the previous section which, however, are more informative since they also mention what has to be avoided.

As another example, if we assume the user preferences

〈*UF*^+^ = *∅*, *UF*^−^ = {*interests*(*music*)}〉

We have that the definition in this section is almost useless and does not provide any recommendation to the user (or, taking another point of view, can produce a huge set of recommendations which include everything but the concert) while the definition in the previous section would produce the solution *S* = {¬*concert*} which is more informative and at least specifies explicitly what has to be avoided, even if it does not provide any positive suggestion.

#### Weakening the definition

Definition 4 (Negative Constraints) can be weakened starting from the idea that a recommendation should be in accordance with most of (rather than all of) the positive preferences avoiding most of (rather than all of) the negative ones. In other words, we can aim at maximising what users like and minimising what they dislike. This corresponds to a weaker interpretation of user preferences, moving from the strong interpretation of definition 4 (Negative Constraints) (where the positive and negative preferences are interpreted as *want* and *hate*) to a weak interpretation, where positive and negative preferences are interpreted as *would like* and *would prefer to avoid* respectively.

##### Definition 5.

**[Support.]** Given a recommendation problem *RP* = 〈*DM*, *OBS*〉, where *OBS* = 〈*UF*, *CXT*〉, where *UF* = 〈*UF*^+^,*UF*^−^〉 distinguishes between positive and negative user preferences. Each set *S* of ground instances of predicates in *R* is a candidate solution with:

- *Positive support* *P*: the subset of *UF*^+^ such that *DM* ∪ *S* ∪ *CXT*⊧*P*
- *Negative support* *N*: the subset of *UF*^−^ such that *DM* ∪ *S* ∪ *CXT*⊧*N*

Among all the candidate solutions, we are interested in those which maximise *P* and minimise *N*. Maximsing and minimising can be interpreted in two ways: (i) in a set-theoretic way using the subset/superset relation to compare two candidate solutions and (ii) in an arithmetic way as maximal and minimal cardinality (we will use the notation ||*X*|| to represent the cardinality of a set *X*).

An ideal solution is one that is maximal in *P* and minimal in *N*, leading to the two following definitions.

##### Definition 6.

**[Weak solutions (set-theoretic).]** Given a recommendation problem *RP* = 〈*DM*, *OBS*〉, where *OBS* = 〈*UF*, *CXT*〉, where *UF* = 〈*UF*^+^,*UF*^−^〉 distinguishes between positive and negative user preferences. A candidate solution *S* with positive support *P* and negative support *N* is a solution to *RP* if and only if for all the other candidate solutions $S^{\prime }$ with positive support $P^{\prime }$ and negative support $N^{\prime }$, we have that $ P^{\prime } \subset P and N^{\prime } \supset N$.

##### Definition 7.

**[Weak solutions (arithmetic).]** Given a recommendation problem *RP* = 〈*DM*, *OBS*〉, where *OBS* = 〈*UF*, *CXT*〉, where *UF* = 〈*UF*^+^,*UF*^−^〉 distinguishes between positive and negative user preferences. A candidate solution *S* with positive support *P* and negative support *N* is a solution to *RP* if and only if for all the other candidate solutions $S^{\prime }$ with positive support $P^{\prime }$ and negative support $N^{\prime }$, we have that $ P^{\prime } <P and N^{\prime }>N$.

Definitions 6 (Weak solutions, set-theoretic) and 7 (Weak solutions, arithmetic) are better suited than Definition 4 (Negative Constraints) in all those cases where there is uncertainty on some user preferences or some of them do not represent a *must* for the user. They collapse to definition 4 (Negative Constraints) if we require that the positive support *P* coincides with *UF*^+^ and the negative support *N* is empty. The definitions above can be too restrictive requiring simultaneous maximisation of positive support and minimisation of negative support. It may be the case, in fact, that there is no solution that simultaneously maximises *P* and minimises *N*. Let us consider for example a situation where there are three candidate solutions:

- *S*_1_: *P*_1_ = {*concert*, *exhibition*}, *N*_1_ = {*sushi*}
- *S*_2_: *P*_2_ = {*concert*}, *N*_2_ = {}
- *S*_3_: *P*_3_ = {*concert*, *exhibition*}, *N*_3_ = {*sushi*, *museum*}

None of them is a solution according to the definitions above. There are different ways of weakening the definitions. A first one is to impose a priority between positive and negative support. In case positive support is prioritized, then we could state that a candidate is a solution if it minimises negative support among those that maximise positive support. This would lead to choosing *S*_1_ above. On the other hand, if negative support is prioritized, leading to choosing the candidates that maximise positive support among those that minimise negative support, then *S*_2_ would be preferred. Alternatively one could characterize a solution as a candidate that maximises the difference *P* − *N*, i.e., the candidates which have the biggest difference between the positive and negative support. Other combinations of *P* and *N* could be optimized (in a set theoretic or arithmetic way given their cardinalities). Or, finally, one could offer all candidates to the user (or at least those that are optimal in at least one of the two dimensions of positive and negative support) accompanying each candidate with its positive and negative support and let the user choose in an informed way. In order to provide more sophisticated discrimination the system should have more information about user preferences, as it will be discussed in the next subsections.

#### Distinguishing between strong and weak preferences

We can also provide an intermediate definition where we partition *UF*^+^ into the subsets *MUST* and *LIKES* and we require that for each candidate the positive support must include all elements in *MUST* and similarly we partition *UF*^−^ into *HATES* and *DISLIKES* and we require that no element of *HATES* belongs to the negative support of a candidate solution.

##### Definition 8.

**[Must and Hates.]** Given a recommendation problem *RP* = 〈*DM*, *OBS*〉, where *OBS* = 〈*UF*, *CXT*〉, where *UF* = 〈*MUST*, *LIKES*, *HATES*, *DISLIKES*〉 distinguishes between strong and weak positive and negative user preferences. A set *S* of literals in *R* is a candidate solution if its positive support *P* is such that $P \supseteq MUST$ and its negative support *N* is such that *N* ∩ *HATES* = *∅*.

A candidate solution is a solution if its positive support also maximises the coverage of *LIKES* and minimizes the coverage of *DISLIKES*.

Regarding the last condition, the same considerations we made at the end of the previous section apply also here: we could put a priority between maximisation and minimisation or adopt other preference/optimization criteria. Let us consider again the example discussed above and consider the user preferences:

*UF* = {*MUST*(*age*(*young*)), *HATES*(*interest*(*food*)), *DISLIKES*(*interest*(*music*))}

If interest in music and food are considered as negative preferences, then definition 4 (Negative Constraints) would not produce any solution. If we adopt a weaker approach then the solution

*S* = {*concert*}covers *MUST* preferences, does not involve *HATES* preferences and could be accepted even though its support is a minimal set {*interest*(*music*)} which is disliked by the user.

#### Grades of preferences and recommendation

The discussion in the previous subsection can be extended by introducing levels of likes and dislikes and then introducing the possibility of having grades of solutions. In particular, let us suppose that positive preferences are partitioned in *n* subsets: *LIKES*_1_,*LIKES*_2_,…*LIKES*_*n*_ where *LIKES*_1_ contains the weakest positive preferences and *LIKES*_*n*_ the strongest ones (*MUST* according to the discussion in the previous subsection). Similarly, negative preferences are partitioned into the subsets *DISLIKES*_1_,*DISLIKES*_2_, …,*DISLIKES*_*m*_ where also in this case *DISLIKES*_1_ contains the weakest negative preferences and *DISLIKES*_*m*_ contains the strongest ones (*HATES* according to the discussion in the previous subsection).

We can thus distinguish among different grades of solutions according to the sets of positive and negative preferences that are covered (not covered).

##### Definition 9.

**[Levels of likes and dislikes.]** Given a recommendation problem *RP* = 〈*DM*, *OBS*〉, where *OBS* = 〈*UF*, *CXT*〉, and *UF* = 〈*LIKES*_1_, *LIKES*_2_,…*LIKES*_*n*_, *DISLIKES*_1_,*DISLIKES*_2_,…,*DISLIKES*_*m*_〉 distinguishes between levels of positive and negative user preferences. A set *S* of literals in *R* is a solution of grade 〈*i*, *j*〉 if its positive support *P* is such that $P \supseteq LIKES_{k}$ for each *k* = *i*…*n* and its negative support *N* is such that *N* ∩ *DISLIKES*_*l*_ = *∅* for each *l* = *j*…*m*.

A solution of grade 〈1,1〉 covers all positive preferences without covering any negative one and corresponds to a solution according to definition 4 (Negative Constraints) and is the most restrictive according to definition 9 (Levels of likes and dislikes). This means that the set of solutions of grade 〈1,1〉 is the smallest one and the sets of solutions for the other grades are supersets of those of grade 〈1,1〉. The solutions of grade 〈*n*, *m*〉 are the less restrictive ones and take into account only what the user *MUST* and *HATES*, which are considered the minimum sets of preferences to be satisfied.

The indices *i* and *j* can be regarded as sorts of sliders that can move independently. Moving *i* from *n* to 1 corresponds to being more restrictive on positive preferences taking into account increasing subsets of these preferences. Moving *j* from *m* to 1, on the other hand, corresponds to being less tolerant on negative preferences taking into account increasing subsets of these preferences.

In the design of a recommender system these sliders could be part of the user interface. The user of the recommender could in this way choose the most appropriate grade of recommendation, given a specific problem and context. The interface could also allow users to decide, for each user preference, the most appropriate level of like and dislike. In this way the preferences that are expressed in a strong and certain way by the user can be distinguished from those that are less strong or less certain (possibly because they are inferred indirectly from user’s behavior). Thus this approach can be regarded as a qualitative way of dealing with uncertainty on user data. In a more sophisticated and parametric interface also the decision on how many grades of positive and negative preferences should be used could be left to the designer.

Let us consider again our running example and suppose we introduce two levels of user preferences and dislikes. As regards positive preferences, we have a strong positive observation *LIKES*_2_ = {*age*(*middle*_*aged*)} and a weak one *LIKES*_1_ = {*interest*(*wine*)}. As regards negative preferences, we do not have strong ones, i.e., observation *DISLIKES*_2_ = *∅*, but we have a weak one *DISLIKES*_1_ = {*interest*(*food*)}. In this case we have two solutions:

- A solution *S*_1_ = {*concert*} of grade 〈2,1〉, whose positive support covers *LIKES*_2_ but does not cover *LIKES*_1_ and whose negative support does not include any *DISLIKE*_*i*_;
- A solution *S*_2_ = {*wine*_*bar*} of grade 〈1,2〉, whose positive support covers all *LIKES*_*i*_ and whose negative support does intersects *DISLIKE*_1_;

In a sense the solutions are not comparable since the first covers all strong user preferences, but does not cover a weak one; however, it also does not involve any negative preferences. In contrast, the second solution covers all positive user preferences at the cost of involving a weak negative one. Other candidate solutions, involving other sets of items are discarded since their grades are lower than the two solutions above.

### Approximate positive preferences

This is the most complex case and amounts to exploiting negative preferences as a sort of approximate positive ones. Let us start from an example and suppose that the language included the user preference *type*_*of*_*food*(*X*) where *X* can assume values in the set {*pizza*, *sushi*, *italian*, *french*}. Let us suppose that the user expresses the negative preference ¬*type*_*of*_*food*(*sushi*). The idea is to interpret this negative observation not only as a negative preference for sushi but also as a positive imprecise information that they would prefer one of the other types of food, that is, as a positive preference *type*_*of*_*food*(*pizza*) ∨ *type*_*of*_*food*(*italian*) ∨ *type*_*of*_*food*(*french*). This preference can then be added to the positive ones and treated in the same way. In this case the definition of solution is as follows:

#### Definition 10.

**[Approximate positive preferences.]** Given a recommendation problem *RP* = 〈*DM*, *OBS*〉, where *OBS* = 〈 *UF*^+^, *UF*^−^, *CXT*〉. Let *UF*^−*N*^ be the complement of the negative observations, that is the set which includes, for each negative preference *p*(*a*_*i*_), the disjunction of the other values that *p* can assume. Let *UF*^*E*^ = *UF*^+^ ∪ *UF*^−*N*^ and let *UF*^*A*^ and *UF*^*C*^ be a partitioning of *UF*^*E*^.

A set *S* of ground instances of predicates in *R* is a solution to *RP* if and only if

- *DM* ∪ *S* ∪ *CXT*⊧*UF*^*A*^
- *DM* ∪ *S* ∪ *CXTisconsistentwithUF*^*C*^ ∪ *NEG* where *NEG* is the negation of *UF*^−^.

In other words, the *complement* of the negative preferences is added to the positive preferences and then the standard definition is adopted. The interesting case in definition 10 (Approximate positive preferences) is the one where the positive preferences generated as disjunction from negative ones (*UF*^−*N*^) are put in the partition *UF*^*A*^ of the preferences to be implied. In this case, in fact, we require that one of the alternative values for a negative preference is implied, that is predicted, in the solution.

This is an active way of using negative preferences which are interpreted as a way of expressing an approximate positive preference. Looking again at the example above, ¬*type*_*of*_*food*(*sushi*) is interpreted as “any type of food is OK, but sushi”. In other words we interpret ¬*type*_*of*_*food*(*sushi*) as a concise way of expressing a positive interest in the other types of food, without a specific strong preference for one of them.

Requiring that one of the other values is implied by the solution corresponds to making an assumption that the set of values is complete, otherwise the requirement may be too strong.

This definition provides more informative recommendations with respect to the case where negative preferences are used as constraints (definition 4 (Negative Constraints)) since it exploits the implicit weak positive preferences derived from the explicit negative ones to provide some positive recommendations while definition 4 uses the negative preference only to cut the set of candidate solutions. As an extreme case if only negative preferences are available the definition in this section can anyway provide some explicit positive recommendations.

It is worth noting that definition 10 (Approximate positive preferences) collapses to 4 (Negative Constraints) in case all disjunctions generated from negative constraints are put into *UF*^*C*^. For definition 10 (Approximate positive preferences) the interesting case is the one discussed above where the disjunctions are put in the partition *UF*^*A*^ of the preferences to be implied. In this way the set of solutions is reduced and thus negative preferences contribute to focus the recommendations.

## Comparison of the approaches

**Fig. 1:**
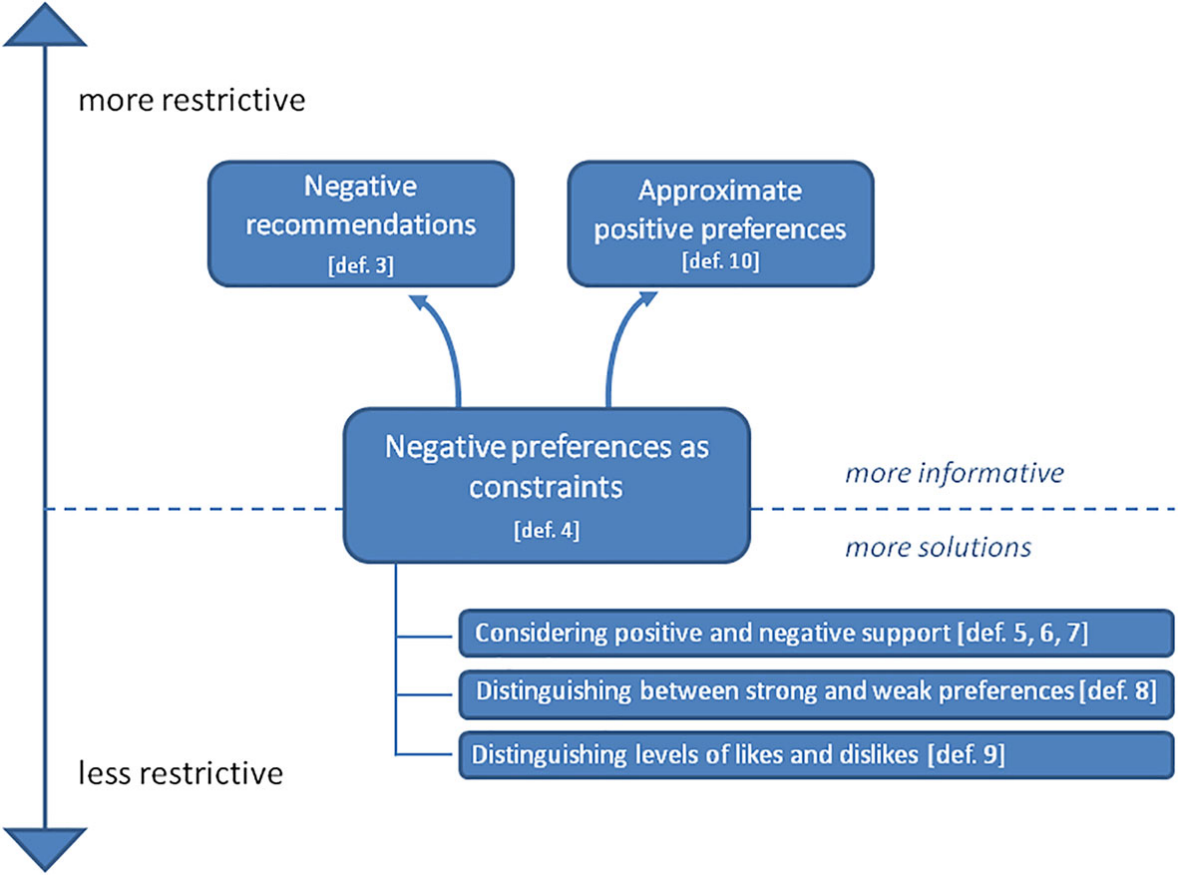
A graphical representation of the proposed approaches for dealing with negative preferences

In this section we analyse and compare the approaches discussed in the previous sections showing that they are related in terms of their restrictiveness. By restrictiveness we mean the dimensions of the sets of solutions that are computed or, from a different point of view, the amount of information provided to users. Figure 1 shows the relations among the approaches.

The constraint (filter) approach of Section 3.2 (definition 4 (Negative Constraints)), which as we will discuss in the next section is the most common in the literature, is in the middle of the graph; the approaches that are above in the graph are more restrictive or informative; those below it are less restrictive.

Definition 3 (Negative recommendations) in Section 3.1 is more informative than definition 4 (Negative Constraints), presenting also negative recommendations - together with the positive ones - to users. This could prove particularly interesting in the infrequent cases where there are many negative preferences and few positive ones. In the extreme case where there are no positive preferences definition 3 (Negative recommendations) can anyway provide some useful recommendations (warning about what should be avoided) while definition 4 (Negative Constraints) is less useful, simply listing everything which is not in conflict with the negative preferences. This does not mean that definition 3 (Negative recommendations) is more restrictive in the sense that it reduces the set of solutions.

On the contrary, definition 10 (Approximate positive preferences) discussed in Section 3.3 is more restrictive than definition 4 (Negative Constraints) because it reduces the set of solutions by using approximate positive preferences. In particular, it removes solutions that are agnostic with respect to the approximate positive preferences generated from the negative ones.

Definition 10 (Approximate positive preferences) provides some positive recommendations making a sort of closed-world assumption, i.e. it assumes that users like the complement of what they dislike, without a strong preference for one of the alternatives. This is the reason why it reduces the set of solutions and clearly this reduction is reasonable (or acceptable) only in case this closed world assumption is reasonable for the specific application and context. An advantage is that in this way it can produce positive answers even when only negative preferences are available.

Definitions 8 (Must and Hates), 6 (Weak solutions (set-theoretic)), 7 (Weak solutions (arithmetic)) and 9 (Levels of likes and dislikes) are less restrictive than definition 4 (Negative Constraints), defining in turn lattices of definitions with different levels of restrictiveness. They produce also solutions that are not in accordance with all positive preferences or that are in conflict with some negative preferences. This may be acceptable or even desirable, especially in cases when the other definitions do not produce any solution or very few solutions. Definition 9 (Levels of likes and dislikes) is the most flexible, leaving the recommender system designer or even the final user the freedom to select the grade 〈*i*, *j*〉 of acceptable solutions, based on the domain feature or even the individual user or case. One can also imagine an iterative and interactive process which starts with restrictive indices and allows users to move them independently and analyse the resulting changes in the set of solutions.

It is worth noticing that the choice of an appropriate definition also depends on the way negative preferences are elicited.

When preferences are collected *explicitly*, users communicate directly what they do not like or hate, for example by rating a set of categories of items presented by the system. Explicit elicitation usually guarantees a high level of certainty about user preferences. On the contrary, with *implicit* elicitation, preferences are inferred from user actions or utterances. For example, we can infer that they do not like something if they never mention or select it. In general, implicit elicitation leads to weaker preferences, possibly with different levels of weakness depending on the user’s actions from which preferences are inferred and from the number/frequencies of actions performed by the user.

Thus, Definition 3 (Negative recommendations) should be used when negative preferences are explicitly elicited, since it implies strong preferences. The same holds for Definition 10 (Approximate positive preferences), especially if it requires that the disjunction of positive preferences generated from negative ones is implied by the solutions, and for Definition 4 (Negative Constraints) in its strongest form. On the contrary, its weakenings (Definitions 6 (Weak solutions (set-theoretic)) and 7 (Weak solutions (arithmetic))) can also be used in connection with implicit elicitation. Similar considerations apply to definitions 8 (Must and Hates) and 9 (Levels of likes and dislikes) which explicitly distinguish between strong and weak preferences and thus can deal with strong explicit negative preferences and weak implicit ones.

## Negative preferences in the literature

In this section we present some relevant recommender systems dealing with negative preferences. This can be seen as an application of our framework to existent cases with the aim to show its usefulness in comparing different approaches. As seen in the Introduction, relatively little work exists that studies how negative preferences can be used to personalize information. In this section, we discuss them according to i) how they deal with negative preferences, and ii) how they gather negative preferences from users. As we will show in the following, to the best of the authors’ knowledge, most recommenders which exploit negative preferences follow the approach we introduced in Section 3.2 (see Definition 4 (Negative Constraints)). However, two notable exceptions can be mentioned: the works by (Zhang et al., 2014) and (Chao et al., 2005), in fact, can be mapped to definitions 3 (Negative recommendations) and 10 (Approximate positive preferences), respectively.

### Negative recommendations

(Zhang et al., 2014) exploit sentiment analysis to extract user opinions about different product features from product reviews. Positive opinions are assigned a + 1 and negative opinions a -1 score. Recommendations and “disrecommendations” (i.e., negative recommendations, as in definition 3) are generated through an Explicit Factor Model approach which uses different matrices to describe users’ numerical ratings on items (as in traditional collaborative filtering approaches), users’ interest in different features (inferred from the number of mentions) and the quality level of all the items for the different features (computed as an average of users’ positive and negative opinions).

### Approximate positive preferences

Adaptive Radio (Chao et al., 2005) is a group recommender which selects songs to play in a shared environment. As suggested by (Jameson & Smyth, 2007), if group recommendations are generated paying attention not to suggest items which will be disliked by any group member (i.e., adopting the so called *least misery* strategy (Masthoff, 2004)), a very subtle differentiation among various degrees of liking may be unnecessary. In such cases, simply concentrating on negative preferences would be more effective^3^. Consistently with these ideas, Adaptive Radio (Chao et al., 2005) actually considers negative preferences as approximate positive ones (see Section 3.3, definition 10 (Approximate positive preferences)). In fact, it implicitly assumes that all songs are acceptable, except if users specify the opposite.

### Negative preferences as constraints

The remaining related work provides examples of recommenders that use negative preferences as constraints on a solution, as in Definition 4 (Negative Constraints).

#### Explicit user preferences

(Pazzani & Billsus, 1997) propose a *content-based* approach hinging on Rocchio’s algorithm (Rocchio & Salton, 1965), which is based on the assumption that most users have a general conception of which documents should be denoted as relevant or non-relevant. Hence, the user’s search query is revised to include an arbitrary percentage of relevant and non-relevant documents as a means of increasing the search engine’s recall, and possibly the precision as well. Starting from this, (Musto et al., 2011) propose to use Random Indexing for dimensionality reduction and an enhanced vector space model (eVSM) to model negative user preferences. The negation operator is used to identify the subspace that will contain the items as close as possible to the positive preference vector and as far as possible from the negative one. MUSICFX (McCarthy & Anagnost, 1998) is a group recommender which considers negative preferences as constraints. MUSICFX suggests radio stations: users are required to provide ratings for all radio stations and they are allowed to express explicitly negative ratings (e.g., a -2 rating indicates that users “hate” a certain radio station). (Koutrika & Ioannidis, 2005) propose an approach for dynamically enhancing a query with user preferences. Here, the degree of interest of a preference is expressed with a positive weight to indicate a positive preference and with a negative weight to indicate a negative preference. Negative, neutral and positive preferences are combined to answer user queries. (Frolov & Oseledets, 2016) propose a method that is able to accurately predict relevant items even from a negative only feedback, in a sort of “users, who dislike that item, do like these items instead” scenario. Finally, (Cena et al., 2018) propose to consider negative preferences in the similarity calculation among users. They exploit Pearson correlations considering not only the overall rating ascribed to a place of interest, but also the aversions expressed by similar users.

#### Sentiment analysis

Systems that resort to sentiment analysis lie at the intersection between explicit and implicit preference elicitation methods. In fact, they rely on contents explicitly written to express users’ opinions; these, however, need to be processed in order to obtain quantifiable preferences. As we anticipated discussing (Zhang et al., 2014), sentiment analysis can be used to highlight fine-grained preferences on specific aspects. In their successive work, (Zhang et al., 2015) also analyse product reviews to extract relevant aspects and aspect-specific sentiment words. As for negative preferences, for users who are particularly interested in a certain aspect, a lower recommendation score will be generated for a product with a bad reputation on that aspect, if compared with users who have different interests. (Musto et al., 2017) observe that multicriteria ratings, although improving the predictive accuracy of the system, can overload users. Thus, they propose a collaborative filtering approach where they automatically extract aspects and the corresponding user sentiment, either positive or negative, from reviews. (Ziani et al., 2017) analyse user reviews by automatically extracting statistical features such as: number of words, emotionalism (proportion of adjectives, adverbs and predicates in comparison with nouns and verbs) or addressing (use of words like “you”). The resulting feature vector is given as input to a semi-supervised support vector machine, which classifies reviews according to their polarity. The corresponding score is then used as a traditional item rating. (Wang et al., 2018) rely on a sentiment-enhanced recommendation algorithm: a preliminary recommendation list is generated through a hybrid recommendation method, Then, it is optimized thanks to sentiment analysis, so that items with many positive reviews are prioritized. (Li et al., 2016) apply sentiment analysis techniques to comments expressed in microblogs to derive frequent program patterns and movie/TV program association rules. Finally, (Priyadharsini & Felciah, 2017) analyse reviews, ratings, and emoticons expressed in e-commerce websites to extract positive, negative and neutral opinions. These are used in a recommender system to suggests positive products. (Tarnowska & Ras, 2021) proposes a novel method to develop a knowledge-based recommender system from text data. It is based on applying an opinion mining algorithm, extracting aspect-based sentiment score per text item.

#### Implicit user preferences

(Lee & Brusilovsky, 2009) implicitly learn user dislikes observing user behaviour: the properties of unsaved objects are added as a negative user profile. Then, the distance between uninteresting and recommended items is computed. Thus, implicit negative preferences make it possible to reliably distinguish ‘good’ items. (Zhao et al., 2018) also consider skipped recommended items as negative preferences, while page views and purchases are considered positive feedback. (Peska & Vojtas, 2013) show how implicit user preferences can be inferred from different actions (e.g., page view, scroll, or time on page), and suggest to consider below average values as negative preferences. They experiment with different aggregation functions and demonstrate that negative feedback can improve recommendation quality. In (Peska, 2017), users are shown a list of recommended items, and those which are not selected are considered as “less preferred” (negative preferences) than those which are selected. Items are then dynamically re-ranked based on “relative preference” relations. Finally, (Paudel et al., 2018) propose a collaborative filtering approach (TC-CML) based on matrix factorization, which models negative preferences using a metric learning framework. Results showed that TC-CML improved recommendation quality on two benchmark datasets, demonstrating the benefit of negative feedback.

#### Explicit and implicit user preferences

Finally, there are systems which use both explicit and implicit user preferences. For example, (Hamed et al., 2012) build two profiles, one for positive and one for negative preferences, consisting in < *genre*, *score* > pairs. Scores under a certain threshold are considered negative preferences. Items are recommended if they are “more similar” to the positive than to the negative profile. Also Social Recommender Systems as a category can use both types of preferences to suggest social media content, people, or both (Guy, 2015; Shokeen & Rana, 2020). In fact, users can provide ratings, tags and comments, which can also express negative opinions. In addition to comments, YouTube collects ratings through a thumbs up/thumbs down widget, while Facebook allows users to state that they are not interested in a certain event or place, even if it does not provide a straightforward way to “dislike” other users’ posts, probably out of netiquette reasons. Some commercial recommenders also adopt similar strategies: Netflix uses a thumbs up/thumbs down widget, while Amazon adopts star-based widgets and radio buttons where some positions are labelled with explicitly negative expressions.

## Evaluation

We mentioned in the introduction that negative preferences have a strong impact on users’ decisions and experiences. Each of the theoretical approaches discussed in this paper can be applied in practice through different algorithms, as it is apparent from our overview of relevant literature, and therefore generate recommendations whose quality depends on specific design and implementation choices. In order to assess our framework, we therefore concentrated on the very concept of “negative-preferences-aware” recommendations, and on the way users perceive them in comparison with recommendations which only take into account positive preferences. Our aim is threefold: firstly, we want to confirm that recommendations which exploit negative preferences are deemed useful; secondly, we want to gain an insight on the user goals and contexts of use which can motivate their adoption; thirdly, we want to understand whether we have missed any relevant ways of using information on negative preferences, from the final user’s point of view.

### Procedure

We devised a scenario-based qualitative study in the restaurant domain, which was chosen because it is connected to our running example and we expect most people to be familiar with it. The study was carried out online due to the COVID-19 pandemic.

Users were asked to imagine that they were alone, on a business trip in a town they had never been to and needed to find a place where they could dine. Going for a stroll to the town center, they could see that many options were available and decided to use a touristic app recommended by their hotel to receive suggestions. They also had to imagine they liked fish cuisine and informal restaurants (style), while they preferred to avoid family-friendly ones (i.e., with children play areas).

Users were presented with four short lists of suggestions, manually generated to signify recommendations considering positive preferences only, and our definitions 3 (Negative recommendations), 4 (Negative Constraints) and 10 (Approximate positive preferences), respectively. The content of the four lists is summarized in Table 1. Notice that the “Positive only” list contains a family friendly fish restaurant (item C, with style in bold), suggested because of the user’s preference for fish, the “Negative recommendations” list explicitly warns users against item C (style in bold, gray background), the “Negative Constraints” list filters out item C, offering only positive suggestions, and the “Approximate positive preferences” list provides additional options, suggesting two restaurants which do not fall into the “informal restaurants” category (items D, E, with style in italic).

**Table 1:**
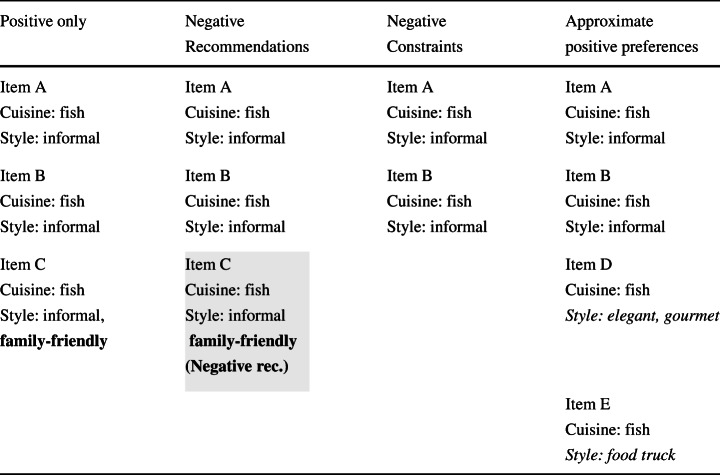
Content of the four recommendation lists

Lists were used as props to encourage reflection. Participants were asked which lists they thought were the most and the least useful, and to provide reasons for their choices. They were explained the rationale underpinning the three lists making use of negative preferences and encouraged to think of additional ways to make use of negative preferences. Finally, they were given the opportunity to provide any further comments.

**Fig. 2:**
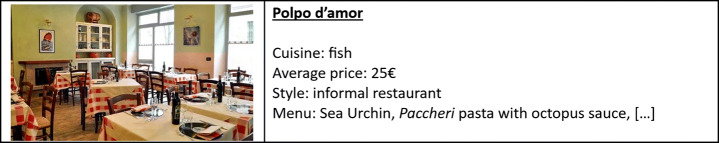
A sample restaurant recommendation

The initial scenario, recommendation lists and questions were presented through web pages implemented using LimeSurvey^4^. For each suggested item, we displayed a photo, the restaurant name and information on its cuisine, style, price range and menu (see Fig. 2). All answers were collected anonimously. We invited colleagues, friends and family members to participate in our study and discontinued data collection when we reached saturation^5^, having involved 20 participants.

### Results

To analyse the collected data, we used a qualitative approached inspired by thematic analysis (Braun & Clarke, 2006). Firstly, we tried to familiarize with the data: we read through a transcript of participants’ answers, observing relevant meanings and patterns and noting potential categories for them. Then, we reread the responses, systematically identified interesting excerpts and labelled them with the previously defined categories. Excerpts were grouped based on the list they referred to and on their overall connotation, either in favour or against it.

No one found that the “Positive only” recommendation list was the most useful. Unsurprisingly, all the participants who chose it as the least useful, or otherwise commented on it, criticized the fact that this list contained items which contrasted with their negative preferences (P2: “It includes things I do not like”, P18: “It suggests items in contrast with my preferences, which is chaotic”).

As far as the other three lists are concerned, participants’ opinions highlight different aspects, depending on the way they conceive choice processes and/or imagined the proposed scenario. The main insights are reported in the following.

#### Negative Recommendations

The list including explicit negative recommendations was appreciated by participants who thought that, in a real context of choice, options other than those presented by the system could also be taken into consideration. In fact, negative recommendations help users to rule out options which could lead to negative experiences (P16: “If I happened to be near a non-recommended restaurant, I could avoid it. If I simply had no information, I could still decide to try it and live an unpleasant experience.”). On the contrary, participants who imagined they would be willing (or forced) to choose only among the listed options found explicit negative recommendations less useful, since they increased the information overload, without representing real opportunities (P19: “Having too much information is confusing. I’d rather receive only useful suggestions”). In this vein, some participants observed that this approach might be appropriate when the overall number of recommendations is limited, or that the user interface should be carefully designed to help users focus (e.g. P16: “[...] clearly separating negative recommendations from positive ones, [...] highlighting reasons for exclusion”).

#### Negative Constraints

The list using negative preferences as constraints is appreciated by participants who would be oriented to make their choice considering only the suggested options. In fact, if the recommender did a good job of filtering, recommendations are accurate, in that they take into account both positive and negative preferences (P8: “This list shows restaurants which correspond to my preferences and doesn’t show restaurants I wouldn’t like to go to”), and in a (more) manageable number (P6: “If there are a lot of (potential) options, it’s better to have a short list to choose from”, P13: “Less options, more accurate”). On the other hand, this solution leaves the least freedom to the user (P12: “The automatic selection made by the system has the highest impact”); consequently, recommendation quality is extremely important.

#### Approximate positive preferences

The list where negative preferences are used to derive approximate positive preferences is appreciated for the variety of suggestions, as it also includes options beyond the user’s exact/explicit preferences (P1: “It gives me more options”, P10: “It suggests exact matches and some more options I might want to consider”, P20: “It allows me to take into account restaurants beyond my preferences. One never knows...”). On the other hand, the greater number of suggestions is considered by some as a negative aspect, since it makes the choice more complex (P14: “There are too many options. I want to use the app to reduce the number of options.”). Furthermore, suggestions that do not reflect explicit preferences are not necessarily appreciated (P5: “There are many options I am not interested in”, P18: “I don’t like street food”).

Participants’ thoughts on additional ways to make use of negative preferences were processed separately, using the same technique. Participants did not suggest alternative approaches for recommendation generation, but mostly proposed features a good recommender should take into account, such as the distance, crowding or atmosphere. A partial exception is represented by one participant who suggested a hybrid approach combining Negative Recommendations and Approximate positive preferences, so as to have more varied options, but with explicit warnings about unsuitable ones. Out of the scope of our framework, but equally interesting to appreciate the importance of negative preferences is the fact that some participants observed that these can vary depending on the moment/context, and that allowing users to explicitly control their negative preferences may therefore be useful (P12: “Dislikes should be contextualized. If you are looking for a restaurant with a patio, at that one time you don’t like restaurants without this facility. A frame or context for the recommendations could be considered”).

## Conclusions

In this paper we showed how negative user preferences can be used in the recommendation process. We presented a comprehensive framework, adopting a logical characterization of the recommendation process. We identified three main conceptual approaches for dealing with negative user preferences, with a set of variations. The formal framework provides a semantics for each one of the approaches, in order to analyse and compare them. Moreover, we showed how the different approaches in the literature fit to our framework, which, on the other hand, suggests alternative ways of exploiting negative preferences which have not been extensively considered in the literature so far. One issue with negative preferences is that, at present, they are less commonly available than positive ones. In addition, traditional recommendation algorithms might be unable to properly manage them. On the other hand, however, we have shown that taking into account information on negative preferences can improve the perceived value of the recommendation service, especially in cases where the cost associated with wrong choices is high. Our evaluation highlighted that the approaches we identified can meet different user needs, situations and ways of conceiving the choice process. In addition, our survey of relevant literature, as well as participants’ thoughts on alternative ways of using information on negative preferences, did not reveal any approach which cannot be covered by our framework; hence, we consider it to be sufficiently comprehensive. With our work, we therefore hope to motivate fellow researchers and designers to gather and exploit information about negative preferences, and to have offered some guidance on how to use it in their recommender systems.

One advantage of our approach regards the explicability of the results. Our model treats explainability as an intrinsic feature: not only the recommendations generated with our approach are explainable, but the model itself is explanation-oriented, due to its relationship with diagnostic approaches (see for example (Hamscher et al., 1992) as an example of the works that inspired our logical characterization of the recommendation process (Cena et al., 2021)).

Moreover, being a parametric approach, it allows to describe different ways of managing negative preferences by manipulating its components. This can be an advantage also to recommender system designers, who can think of novel systems by reasoning on how to deal with negative preferences.

The analysis in the paper and the fact that we have a common semantics for all definitions could provide guidelines to recommender system designers who are interested in dealing with negative user preferences. Different dimensions, in particular, can be taken into account:

- The type of negative information which is available and the way it is elicited can provide a first discrimination among the approaches, as discussed in Section 4.
- The type of recommendation to be provided can suggest the designer whether providing negative recommendations can be useful in the specific application or whether complex recommendations as those provided by definition 9 (Levels of likes and dislikes) are suitable for the user.
- The type of recommendation to be provided can suggest whether weak solutions that do not take into account the totality of user preferences are acceptable. In some domains or applications this may be unacceptable.
- The cost associated with users selecting unsuitable options (e.g., in terms of money, time and consequences) should be considered when deciding whether to provide negative recommendations: for example, letting users listen to a song they are unlikely to appreciate is probably less serious than letting them make a “wrong” purchase or dine in a terrible restaurant.
- Information on the type of available knowledge and its completeness is relevant for choosing definition 10 (Approximate positive preferences), which is suitable if we can assume that the model is exhaustive with regards to the user preferences for which we adopt this approach.
- Section 4 provides a comparison of the definitions according to how restrictive they are. Restrictiveness can be an advantage in terms of being more informative towards the user but it is appropriate if strong negative preferences can be available and it is reasonable to assume completeness of the model or, from a different point of view, assuming that recommendations to the user are provided at the best of what is modeled.
- The user need for transparency should be considered, since high transparency helps to trust the system. As seen in the introduction, highlighting negative recommendations can help to strengthen user trust when only few positive recommendations are available.

Logic is used as a simple unifying framework allowing us to provide a ground to analyse and compare the alternative approaches in an intuitive way. The definition can be used as a reference to decide if and how to deal with negative preferences in a recommender which can then be implemented in different ways. As we noticed, there are efficient techniques to implement the definition we provided, dealing also with forms of incompleteness and uncertainty in the domain model and in user data (see (Hamscher et al., 1992)). On the other hand, a limitation deriving from the use of logic is that it can only deal with qualitative forms of incompleteness and uncertainty in the model and cannot deal with quantitative forms such as those expressed using numeric approaches. We plan to extend our work in this direction, following again the road traced by other formalizations of problem solving tasks such as diagnosis (see again (Hamscher et al., 1992)).

## Data Availability

This manuscript has no associated data.

## Footnotes

1 http://youtube.com

2 http://facebook.com

3 Notice that, even though according to (Quijano-Sanchez et al., 2013) *least misery* is one of the most popular strategies for group recommenders (see e.g., (Recio-Garcia et al., 2009; O’Connor et al., 2001)), most of them do not collect negative preferences.

4 https://www.limesurvey.org/

5 Saturation can be defined as the point at which “additional data do not lead to any new emergent themes” (Given, 2015) and is therefore used as a guidance for data collection/analysis in qualitative research
